# Lower constraint testing enhances the testing effect for some contextual details but not others

**DOI:** 10.1002/brb3.3380

**Published:** 2024-01-09

**Authors:** Konstadena L. Giannakopoulos, Matthew P. McCurdy, Allison M. Sklenar, Andrea N. Frankenstein, Pauline Urban Levy, Eric D. Leshikar

**Affiliations:** ^1^ University of Illinois at Chicago Chicago Illinois USA

**Keywords:** context memory, item memory, location memory, retrieval practice, spatial memory, test constraints, testing effect

## Abstract

**Introduction:**

Retrieval practice has been shown to be an effective means of learning new information, a memory phenomenon known as the *testing effect* or the *retrieval practice effect*. Some work suggests that the magnitude of the testing effect can be enhanced when the test used for retrieval practice uses fewer cues to retrieve previously studied information. It is unclear, however, whether such testing benefits extend to peripheral contextual details associated with studied materials (e.g., location where stimuli appear, font color in which items are presented, etc.). In this experiment, we examine both item memory (i.e., memory for the studied items) and context memory under conditions where the intervening test offers fewer cues (i.e., lower constraint) compared to more cues (higher constraint) to better understand item and context memory testing effects.

**Methods:**

Participants first studied word pairs presented in one of eight locations as well as in either red or green font color. Then, in the re‐exposure phase, participants processed materials in two types of intervening tests (lower constraint and a higher constraint test) as well as in a restudy condition, before a final memory test.

**Results:**

For item memory, results showed that memory was better in the lower constraint testing condition compared to both the higher constraint testing condition as well as the restudy (control) condition. For context memory, results indicated improved memory for location context under lower constraint testing compared to both higher constraint testing and restudy conditions. There was no difference in memory, however, for color context across all conditions.

**Conclusion:**

Overall, these findings suggest that providing fewer cues to aid retrieval in the intervening test can induce better memory for both items as well as some contextual details.

## Graphical abstract

Research has shown that testing is an effective strategy for improving memory for previously learned material compared to restudying (i.e., the testing effect or the retrieval practice effect). In this investigation, we examine whether providing less information about target items during a practice test (a lower constraint test) improves item and context memory over a practice test with more information about target items (a higher constraint test). Results showed that the lower constraint practice test led to better memory relative to the higher constraint test, restudy, and control conditions. Overall, we establish a way to increase the magnitude of the testing effect in memory, which may be a way to optimize memory using this well‐known study strategy.

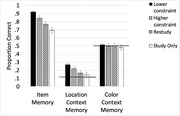

## 1 INTRODUCTION

The testing effect (also known as the retrieval practice effect) is the memory phenomenon where memory is improved for materials that are tested (i.e., retrieved) compared to materials that are simply restudied (Roediger & Karpicke, 2006; Rowland, 2014). Substantial evidence suggests that testing is a powerful way to improve memory for studied materials (Adesope et al., 2017; Rowland, 2014). In a typical testing effect experiment, participants engage in three phases: an initial study or encoding phase, a re‐exposure phase where materials are restudied or tested with an intervening memory test, and a final memory test phase. The testing effect is a robust memory phenomenon shown to improve memory for a variety of different types of materials such as word pairs (Carpenter, 2009, 2011), single words (Szpunar et al., 2008), and educational texts (Dirkx et al., 2014), among others. Our primary goal in this investigation is to examine how changes in the re‐exposure phase (e.g., how participants process previously studied items) affect both item memory (memory for studied items) as well as context memory (memory for contextual details associated with studied items) to better understand the extent that testing improves memory for materials compared to control conditions (restudy, study‐only [e.g., items that are studied, but not shown again during the re‐exposure phase]).

Past work has demonstrated that different types of tests used in the re‐exposure phase influence how well‐studied items are remembered on a final test. For example, work has shown that using recall tests in the re‐exposure phase leads to better memory compared to using recognition tests (Butler & Roediger, 2007; Carpenter & DeLosh, 2006; Glover, 1989). The rationale behind this effect (i.e., differences in types of intervening tests in the re‐exposure phase affect the magnitude of the testing effect) is that different types of tests affect how participants access or retrieve previously studied information, and those differences, in turn, affect how well that information is retrieved on a final test. In line with the idea that the type of intervening test influences memory, work from one investigation examined the extent *testing constraint* (i.e., tests that differed in how constrained participants were in producing previously studied items) in the re‐exposure phase affected memory on a final test (Giannakopoulos et al., 2021). In that investigation, participants studied word pairs (e.g., open–close) in the initial study phase. Then, in the re‐exposure phase, participants tried to retrieve studied items in a lower constraint test where participants were given fewer retrieval cues to retrieve studied items (e.g., open–c___; participants were only given the first letter of the target word), as well as a higher constraint test where participants were given more retrieval cues to retrieve studied items (e.g., option–csloe; participants were given all the letters of the target word in a scrambled order), and a restudy condition where they were simply re‐exposed to studied items (e.g., open–close). Results indicated that items processed in the lower constraint test led to improved memory for studied items compared to both the higher constraint test condition as well as the restudy condition. Such findings are consistent with past work showing that tests that provide fewer retrieval cues in the re‐exposure phase often induce improved memory performance on a final test (Butler & Roediger, 2007; Carpenter, 2009, 2011; Carpenter & DeLosh, 2006). In this investigation, we further examine the extent that different types of tests in the re‐exposure phase, especially those that induce lower compared to higher testing constraints, affect memory to better understand conditions under which the magnitude of the testing effect varies.

Although there is a rich body of evidence showing that testing enhances memory, the majority of this work has focused on item memory, or memory for the materials studied in an initial learning phase (e.g., such as studied words). Much less work, however, has investigated the extent that testing might also improve memory for contextual details (i.e., details that accompanied the episode, such as in which location stimuli were presented, or in which font color stimuli were presented). Intriguingly, theoretical work suggests that context memory may play a critical role in the testing effect. Specifically, the episodic context account (Karpicke et al., 2014; Whiffen & Karpicke, 2017) suggests that a primary mechanism underlying the testing effect (or retrieval practice effect) is related to encoding context at initial study, and then retrieving/reinstating that context during the re‐exposure phase. This account suggests that the process of encoding and then retrieving/reinstating contextual details in the re‐exposure phase allows for easier access to studied materials, which results in improved memory for tested compared to restudied items on a final test. If it is the case that retrieving context in the re‐exposure phase contributes to the testing effect, then one implication of the episodic context account is that context memory should be better for materials processed in an intervening test compared to restudy conditions. A look through the limited studies that have investigated context memory in testing effect procedures, however, shows mixed results, with some studies showing context memory improvement for tested compared to restudied materials (Akan et al., 2018; Brewer et al., 2010, Experiment 2), whereas others have not (Brewer et al., 2010, Experiment 1; Hong et al., 2019; Smith et al., 2019). It is worth noting that in each of these investigations, however, context memory was measured for only one contextual detail (location, color, list membership, etc.). To gain a richer understanding of the extent that context memory improves for tested compared to restudied materials, it is worthwhile to assess memory for more than one contextual detail within the same experiment to better understand potential context memory testing effects. In this investigation, we assess context memory for two different details (location, font color of presented stimuli) to advance understanding of the extent context memory improves for tested compared to restudied materials. We are particularly interested in the extent context memory testing effects are larger for materials processed in a lower compared to a higher constraint intervening test. Finding improved context memory for materials studied in lower compared to higher constraint intervening tasks would be in line with the episodic context account, and further consistent with past work showing that the magnitude of improvement in memory from testing is greater when the intervening test (i.e., re‐exposure phase) uses fewer cues to aid retrieval (Carpenter & DeLosh, 2006).

In this investigation, we examine the extent item and context memory would be better for materials processed in a lower compared to a higher constraint intervening task. We also examine the extent that item and context memory would improve for items processed in testing conditions (lower, higher constraint) relative to nontested control conditions (restudy, study‐only). In this experiment, participants first studied word pairs presented in one of eight locations on a screen in two different font colors. Then, in the re‐exposure phase, participants processed materials in a restudy condition, as well in both lower and higher constraint test conditions, before then completing a final memory test assessing both item and context memory (location context, color context). We have two predictions in this experiment. First, for item memory, we expect to see better memory for materials processed in lower compared to higher constraint conditions. Such a finding would be aligned with past work showing that intervening tests that offer fewer cues to help participants retrieve studied materials are typically associated with improved memory on a final test (Carpenter & DeLosh, 2006). We also expected that item memory would be better in both testing conditions (lower, higher constraint test) compared to control (restudy, study‐only [i.e., items that are studied, but not shown again during the re‐exposure phase]), in line with past work on the testing effect (Roediger & Karpicke, 2006; Rowland, 2014). Second, for context memory, we expect better memory for materials processed in both testing conditions (lower, higher constraint) relative to restudy, which would be consistent with past work showing context memory testing effects. We also expect that context memory will be better for materials processed in the lower compared to the higher constraint test conditions, which is in line with the idea that intervening tests that use reduced cues lead to bigger improvement in memory, and further support the important role of context memory theorized to underlie the testing effect in memory (Karpicke et al., 2014). Overall, results of this experiment will advance understanding of the testing effect for both item and context memory.

## 2 METHODS

### 2.1 Participants

Sixty‐two participants were recruited; however, a total of 15 participants were removed from analyses.^1^ Thus, data reported reflect 47 University of Illinois at Chicago undergraduate students (age *M* = 19.00, *SD* = 1.30, range = 18–23, 27 females) recruited from the introductory psychology course subject pool. A priori power calculations were computed in G*Power (Faul et al., 2009) using an effect size based on our prior work on the extent lower and higher constraint tasks affect memory (*d* = 0.55; McCurdy et al., 2017, 2019). Results showed that we needed 44 participants to achieve .80 power to detect our effect of interest. Because we included materials portrayed in red and green fonts, we only recruited participants who were not colorblind. All participants gave their written informed consent in accord with the University of Illinois at Chicago Institutional Review Board and were given course credit for their participation.

### 2.2 Materials

A total of 96 cue–target word pairs were chosen from the University of South Florida word association norms (Nelson et al., 2004). All word pairs consisted of highly associated cue–target pairs (e.g., open–close; forward cue–target associative strength [FSG] > .50, Mean FSG = .624). Each cue and target word contained between four and seven letters, as done before (Giannakopoulos et al., 2021). Across participants, word pairs were counterbalanced to create a total of eight versions such that each word pair occurred exactly once in each condition (lower constraint test, higher constraint test, restudy, study‐only) and presented equally in both red and green font. Because each word pair appeared in each experimental conditions across participants, this means that all conditions used stimuli that consisted of the same cue–target associative strength. As for the location, word pairs were programmed to appear in one of the eight locations in a nonrepeating order.

### 2.3 Procedure

**FIGURE 1:**
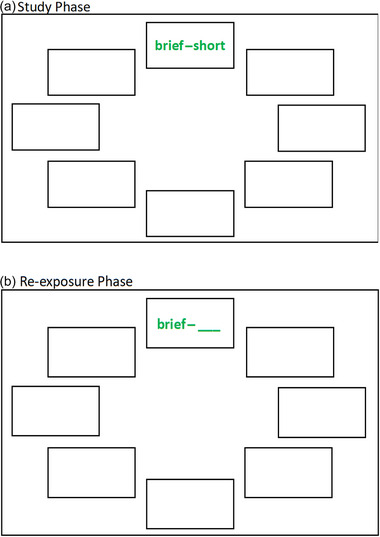
Trial schematic depicting a study phase and re‐exposure phase trial. In the (a) study phase, the word pair was shown in one of the eight locations, each equally distanced from the center, and in red or green font color. In the (b) re‐exposure phase, stimuli were presented in the same location and same font color as the study phase. The example depicts a lower constraint test trial.

The current experiment consisted of three phases (study phase, re‐exposure phase, final test phase) completed over two sessions spaced 2 days apart (48 h). Day 1 consisted of the study phase and the re‐exposure phase, whereas Day 2 consisted of the final test phase. All three phases were presented using E‐Prime software (Psychology Software Tools, 2012). On Day 1, participants first completed training on shortened versions of the study phase and the re‐exposure phase (six trials each) prior to the experiment to ensure they understood task instructions. The word pairs used during training did not appear elsewhere in the experiment. During Day 1, participants were unaware that there was a final memory test (on Day 2) of the target items or that they would be asked to remember contextual details associated with target items (i.e., incidental learning procedure).^2^ After training, participants began the study phase, where participants were shown a total of 96 word pairs, one at a time, at a fixed pace of 5 s per trial with a 500‐ms fixation between each trial. Figure 1 shows an example trial depicting what participants were shown during the study phase. The word pairs were presented in one of eight locations in a randomized, nonrepeating order, and further, half the word pairs were presented in red font and the other half in green font, in a randomized order.

After completing the study phase, participants moved on to the re‐exposure phase. In a within‐subject design, participants processed stimuli in two types of intervening tests (lower constraint, higher constraint) and a restudy opportunity in the re‐exposure phase, as done before (Giannakopoulos et al., 2021). In the re‐exposure phase, participants saw 72 (of the 96 total) word pairs in a different random order from the study phase. The remaining 24 word pairs did not appear in the re‐exposure phase and served as study‐only control trials, as done before (Akan et al., 2018). Figure 1 shows an example trial of the re‐exposure phase. During this phase, word pairs were presented in the same font color and the same location as at study. Word pairs were shown across three conditions: lower constraint test, higher constraint test, and restudy (24 pairs per task). The re‐exposure phase consisted of six blocks (two blocks per condition) of 12 trials each. All trials within a block were of the same condition (e.g., lower constraint test). Across participants, blocks were randomized with the exception that the same condition was not performed in consecutive blocks. Participants were notified between blocks which condition they were about to perform. All trials were self‐paced and separated by a 500‐ms fixation. In the lower constraint condition, participants were presented with a cue word from the study phase followed by a blank line (e.g., open–___). Participants were instructed to recall the paired target word from the study phase and type both words on the screen, one at a time. This procedure (typing both the cue and target word in all conditions) was implemented to ensure participants were processing both the cue and target word. In the higher constraint condition, participants were presented with both the cue and target word of a word pair, but the target word was scrambled (e.g., option–csloe). Participants were instructed to unscramble the letters to retrieve the target word, and then type out both words on the screen, which is a procedure used before (Giannakopoulos et al., 2021). The lower constraint and higher constraint intervening tests were analogous to procedures used in past work (Giannakopoulos et al., 2021; McCurdy & Leshikar, 2022; McCurdy et al., 2017, 2019, 2021; McCurdy, Frankenstein, et al., 2020; McCurdy, Viechtbauer, et al., 2020). In the restudy (control) condition, participants were presented with an intact word pair from the study phase (e.g., open–close) and were asked to type both words. No feedback was given in any condition in the re‐exposure phase, similar to previous testing effect investigations (Akan et al., 2018; Giannakopoulos et al., 2021; Rowland, 2014). After the re‐exposure phase, participants were instructed to return to the laboratory exactly 48 h (2 days) later and were dismissed.

**FIGURE 2:**
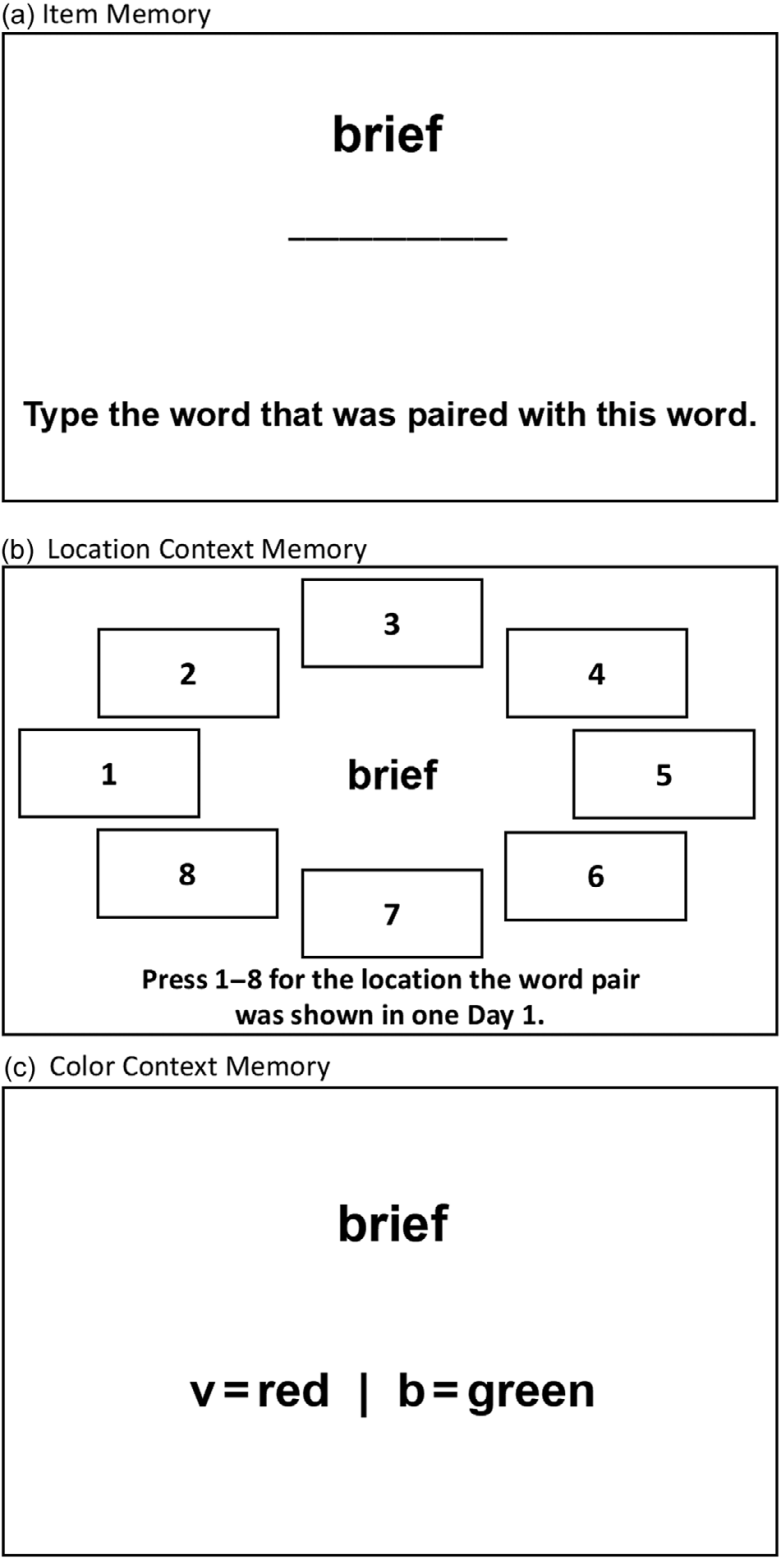
Example trial schematic of day 2 (final test phase) of the experiment for each memory measure (item, location context, color context).

After a 48‐h delay, participants returned to the lab for the final test phase. In this phase, participants were given a cued recall test to assess item memory and a recognition test to measure context memory for location and color. An example final test phase trial is shown in Figure 2. Before completing the final test, participants were first trained on a shortened version of the final test phase to ensure they understood task instructions. After training, participants were then shown each of the 96 *cue* words, one at a time, in a different random order from Day 1, presented in a white font on black background. For our item memory measure, we used a cued recall task where participants were asked to type the word that was paired with the presented cue word from Day 1. Participants were instructed to leave the response blank if they were unable to recall the target word. After giving their item memory response, participants then completed the context recognition test assessing both location and color context memory. For location context, participants were shown eight location boxes labeled 1 through 8 and were asked to type the number corresponding to the location in which the word pair was presented in the study phase of the experiment. Participants were then asked how confident they were in their location context memory choice, on a scale of 1–6 (1 = *not at all confident*, 6 = *extremely confident*; inspired by a similar procedure, Carpenter, 2011). Next, for color context memory, participants were asked to judge in which font color (red or green) the word pair appeared in the study phase of the experiment by pressing the 1 or 2 key on the keyboard (1 = *red*, 2 = *green*). Similar to the location context, participants then rated how confident they were in their color context memory response using the same scale of 1–6 (1 = *not at all confident*, 6 = *extremely confident*). After completing the final test phase, participants were debriefed and dismissed.

## 3 RESULTS

In this section, we report the data from the re‐exposure phase as well as the final test phase. In the re‐exposure phase, participants accurately produced items in the restudy condition (*M* = .99, *SD* = .07). For the two practice test conditions, participants more accurately recalled items in the higher constraint test (*M* = .94, *SD* = .24) than the lower constraint test (*M* = .83, *SD* = .38), *t*(46) = 4.25, *p* < .001.

Turning to the final test data, item memory (cued recall) performance was calculated as a proportion of items correctly recalled in the final test phase out of the total number of items correctly produced in the re‐exposure phase, as done before (Giannakopoulos et al., 2021; Runquist, 1983). Location memory performance was calculated using a conditional measure that reduces the influence of item memory performance (Murnane & Bayen, 1996), as done in past work (Leshikar & Gutchess, 2015; Leshikar et al., 2016; Leshikar, Dulas, et al., 2015). Specifically, location context memory was calculated as the proportion of correctly recalled items (i.e., item hits) that were also correctly identified with their location (location hits/item hits). Using an analogous procedure, color context memory was calculated as the proportion of correctly recalled items that were also correctly identified with their font color (color hits/item hits). For each memory measure (item, location context, color context), we ran a repeated‐measures analysis of variance (ANOVA) and then ran post hoc paired *t*‐tests corrected for multiple comparisons across the four conditions (lower constraint test, higher constraint test, restudy, and study‐only) when appropriate.

**FIGURE 3:**
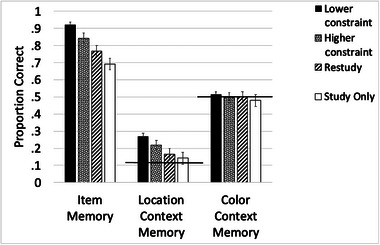
Final memory performance across all four conditions for each type of memory estimate used in this experiment (item, location context, and color context). Item memory exhibited the standard testing effect (e.g., lower and higher constraint > restudy and study only), and further, materials processed in the lower constraint condition yielded the highest memory performance relative to all other conditions. Similarly, location context memory showed the standard testing effect, and further, materials processed in the lower constraint condition led to the highest memory performance relative to all other conditions. For color context, there were no significant differences across conditions. Such results suggest that testing conditions that use fewer cues during the re‐exposure phase yield improved item and location context memory. Item, location, and color context memory estimates reflect conditional estimates of memory as described in the main text. For both contextual details, the level of chance is marked.

First, we examined item memory. We found significant differences among the four conditions, *F*(3, 46) = 50.52, *MSE* = .045, *p* < .001, *η* ^2^ = .52, as shown in Figure 3. Follow‐up analyses corrected for multiple comparisons revealed that the lower constraint test (*M* = .92, *SD* = .08) led to better memory compared to both the restudy condition (*M* = .76, *SD* = .15), *t*(46) = 6.89, *p* < .001, *d =* 1.01, and the study‐only condition (*M* = .69, *SD* = .16), *t*(46) = 9.61, *p* < .001, *d =* 1.40. Similarly, the higher constraint test (*M* = .84, *SD* = .13) led to better memory compared to both the restudy, *t*(46) = 4.73 *p* < .001, *d* = 0.69, and study‐only conditions, *t*(46) = 8.44, *p* < .001, *d* = 1.23. Importantly, there was also a significant difference between the two practice test conditions, where the lower constraint test led to better item memory than the higher constraint test, *t*(46) = 4.37, *p* < .001, *d* = 0.64. This finding supports our prediction that fewer test constraints yield greater memory benefits from testing, in line with prior work (Carpenter, 2009; Giannakopoulos et al., 2021; Glover, 1989).^3^

For location context memory, we found significant differences among the four conditions, *F*(3, 46) = 16.46, *MSE* = .022, *p* < .001, *η* ^2^ = .26, as shown in Figure 3. Follow‐up analyses corrected for multiple comparisons revealed that the lower constraint test (*M* = .26, *SD* = .16) led to better recognition memory than both the restudy (*M* = .17, *SD* = .08), *t*(46) = 4.40, *p* < .001, *d* = 0.64, and study‐only conditions (*M* = .14, *SD* = .06), *t*(46) = 5.35, *p* < .001, *d* = 0.78. Similarly, the higher constraint test (*M* = .22, *SD* = .11) led to better memory than both the restudy, *t*(46) = 2.86, *p* = .02, *d* = 0.42, and study‐only conditions, *t*(46) = 4.60, *p* < .001, *d* = 0.67. We also found a significant difference between the control conditions, where memory was better in the read condition than the study‐only, *t*(46) = 2.13, *p* = .039, *d* = 0.31. Critically, we observed a difference between the testing conditions, where memory was better in the lower compared to higher constraint condition, *t*(46) = 2.30, *p* = .031, *d* = 0.33.^4^

For color context, we found no significant differences between the four conditions, *F*(3, 46) = 0.69, *p* = .562, as shown in Figure 3.^5^

## 4 DISCUSSION

The main purpose of this investigation was to examine item and context memory effects for materials tested in lower and higher constraint test conditions as well as control conditions (restudy, study‐only). We have two primary results. First, we found improved item memory for items processed in the lower compared to higher constraint conditions. Such a finding is consistent with past work demonstrating that different memory effects emerge from different varieties of intervening tests (Butler & Roediger, 2007; Glover, 1989) and is further aligned with work showing that tests that offer reduced retrieval cues tend to lead to enhanced memory (Carpenter & DeLosh, 2006). We also found a standard item memory testing effect where tested items (both lower and higher constraint conditions) led to improved memory compared to control conditions (restudy, study‐only). Second, and most importantly, we found that context memory improved for materials processed in lower compared to higher constraint test conditions, as measured by location context, but not color context. Such a finding suggests that materials processed in lower constraint testing conditions yield improved memory for some contextual details relative to higher constraint tests. We also observed improved context memory for tested materials (lower, higher constraint conditions) compared to control (restudy, study‐only conditions) for location context memory, but not for color context memory. Such a result partially supports the episodic context account of the testing effect (Karpicke et al., 2014; Lehman et al., 2014; Whiffen & Karpicke, 2017). Overall, results of the current investigation add to the literature on the mnemonic benefits of testing (e.g., retrieval practice) on memory.

In this investigation, we found improved item memory for materials processed in the lower compared to higher constraint condition. Finding improved memory for materials in the lower compared to higher constraint test condition is further consistent with past work demonstrating that tests that use fewer cues to guide retrieval during re‐exposure lead to enhanced item memory (Carpenter & DeLosh, 2006). This finding adds to the literature suggesting that the type of test used in the re‐exposure phase has a strong effect on the magnitude of the testing effect in memory (i.e., how much memory improves for tested materials compared to control). Interestingly, our item memory results (improved memory for lower compared to higher constraint test conditions) are consistent with another finding in a different memory domain, the generation effect (Slamecka & Graf, 1978). Over numerous investigations, prior work has shown that memory for materials produced in lower constraint generation tasks (e.g., producing a target word from the prompt, open–c___) is significantly better than materials generated in higher constraint generation tasks (e.g., open–csloe; McCurdy & Leshikar, 2022; McCurdy et al., 2021; McCurdy, Viechtbauer, et al., 2020). In particular, this past work on the generation effect has demonstrated that producing materials under lower compared to higher constraint conditions may act to increase relational processing of the cue–target relationship of word pairs, and that this enhanced relational processing leads to improved memory for materials produced in lower compared to higher constraint conditions (McCurdy, Frankenstein, et al., 2020). Future work may be necessary to understand whether a similar improvement in relational processing for items encountered in lower compared to higher constraint tests in testing effect procedures may be contributing to item memory testing effects. In addition to improved item memory for lower versus higher constraint conditions, we also found that item memory was better for items processed in both testing conditions (lower, higher constraint) compared to controls (restudy, study‐only), which is consistent with a vast literature showing that testing (or retrieval practice) improves item memory (Adesope et al., 2017; Glover, 1989; Roediger & Karpicke, 2006; Rowland, 2014). Finding ways to improve memory is an important scientific endeavor (Bjork & Bjork, 2011; Burden et al., 2021; Frankenstein et al., 2020, 2022; Ilenikhena et al., 2021; Kadwe et al., 2022; Leshikar, In Press; Leshikar et al., 2019, 2017; Leshikar, Park, et al., 2015; Matzen et al., 2015; Patel et al., 2022; Sklenar et al., 2022, 2021, 2023; Udeogu et al., 2022; Urban Levy et al., 2023; Villasenor et al., 2021), and the results of this investigation add to that cause.

One of our primary goals in this experiment was to investigate context memory testing effects. We did so using a procedure that assessed context memory for more than one contextual detail, which is an advance over past work that has only used a single measure of context memory (location: Akan et al., 2018; Brunyé et al., 2020, font color: Giannakopoulos et al., 2021; Hong et al., 2019, list membership: Smith et al., 2019, voice source: Brewer et al., 2010, etc.). Results of the current investigation showed that context memory for location was better for materials processed in both testing conditions (lower, higher constraint) compared to controls (restudy, study‐only), but not for color context memory. Such results give partial support to the episodic context account of the testing effect (Karpicke et al., 2014). The episodic context account posits that people encode contextual information during initial learning/study, and then retrieve/reinstate that context during an intervening test. The episodic context account further suggests that this process of encoding and then retrieving contextual information leads to improved memory on a final test because such information is more accessible or retrievable on the final test. The fact that we found improved recognition memory for location context, but not color context, fits with the limited past work in the domain, where some studies have found context memory testing effects (Akan et al., 2018; Brewer et al., 2010, Experiment 2), whereas others have not (Giannakopoulos et al., 2021; Hong et al., 2019; Smith et al., 2019). More work may be necessary to understand the conditions under which context memory improves under testing effect procedures. Importantly, we also found improved location context memory for materials encountered in the lower compared to higher constraint conditions. This result suggests that context memory improves in testing conditions that use fewer cues as part of the testing procedures, at least as measured by location context. Overall, results of this investigation expand our understanding of context memory testing effects, which is an advance in understanding how episodic memory is modulated by testing (e.g., retrieval practice).

In this investigation, we found context memory testing effects for location, but not for color context, which does not fully support the episodic context account of the testing effect.

A closer look through the studies that have examined context memory in testing effect procedures, however, shows that the studies that have found context memory effects have used procedures where there is a richness to the contextual detail they are measuring (e.g., eight different possible locations where studied items can appear; Akan et al., 2018), whereas for studies that have not shown context memory testing effects, the contextual detail they are measuring is less rich (e.g., two lists, Smith et al., 2019; two to four color options, Giannakopoulos et al., 2021; Hong et al., 2019). This is relevant because another suggestion of the episodic context account suggests that participants use contextual information they are able to retrieve to limit, or restrict, the search set, which allows them to more readily access memory for individual items (Karpicke et al., 2014). Thus, it may be that when there is more variety for a given contextual detail (such as items appearing in one of eight locations), this allows participants to restrict their search to only those items that shared that contextual feature, which promotes access to that information leading to improved memory. By contrast, when contexts have fewer features that can be used to distinguish items (such as “only” two colors), this may reduce participants’ ability to restrict the memory search set, leading to a reduced likelihood of accurately retrieving contextual details.

Thus, it may be that context memory is generally improved under testing effect procedures, but that the effects are more readily observable under conditions where the context has sufficient variability to help make encoded information more distinctive from one another. The idea that contextual details can be used to restrict the search set in memory, thereby making items more distinctive leading to enhanced memory, is consistent with past theoretical and empirical work (Raaijmakers & Shiffrin, 1981; Wixted & Roehrer, 1993, 1994). Turning back to our location and context memory findings, it may be that we found improved location context, but not color context memory, because participants were better able to restrict their search set for the contextually richer location detail relative to the sparser color context detail. Future work might further investigate this possibility to better understand the extent context memory might be an important mechanism underlying the testing effect in memory.

Evidence from this investigation showed that materials encountered in lower constraint testing conditions improved memory for various episodic details (item memory, location context memory) relative to higher constraint testing. Such findings have the potential to easily translate into educational settings. Specifically, it may be that devising practice tests that provide fewer retrieval cues (i.e., lower constraint testing conditions) during practice tests might lead to higher retention of course materials. Understanding how different varieties of encoding or study strategies influence memory is an important area of research (Colton et al., 2013; Dunlosky & Hertzog, 2000; Finley & Benjamin, 2012; Hertzog et al., 2008; Jackson et al., 2019; Leshikar & Duarte, 2012, 2014; Leshikar et al., 2010; Leshikar, Dulas, et al., 2015; Meyer et al., 2020; Naveh‐Benjamin et al., 2007; Tullis et al., 2013; Wong et al., 2017), and the present investigation adds to the literature on techniques to improve memory for studied materials.

Although we found evidence that materials processed in lower compared to higher constraint conditions improved both item and location context memory, there are three limitations of the current investigation that warrant further discussion. First, in our primary memory analyses, we used conditionalized estimates of memory including only those trials that were successfully produced in the re‐exposure condition. The downside of this approach, however, is that it may be that some items were correctly produced during the re‐exposure phase because they were inherently more memorable (and not because of the experimental condition in which they were presented), which is a phenomenon known as the item selection effect that can impact memory performance. We tried to work against possible item selection effects by using stimuli that were carefully controlled (e.g., all high associate cue–target pairs). Further, because all stimuli (i.e., word pairs) were counterbalanced to appear in all experimental conditions, we think any possible item selection effects should have cancelled out across participants. Indeed, given that analyses of both the conditionalized and the unconditionalized memory data for both item and location context were highly similar, we think it is unlikely that item selection effects strongly affected our results. Second, because we used high associate cue–target pairs as stimuli, it is possible that this allowed participants to successfully guess the target associated with the cue during both the re‐exposure as well as the final test phase even in instances where they could not explicitly recall the correct target (e.g., given the cue “open,” it is possible that participants might correctly guess the target as “close”). Because we counterbalanced all stimuli (i.e., word pairs) to appear in all conditions across participants, however, any effects of guessing should cancel out, and thus, should not strongly affect our results. Third, although we found no memory effects for the color context memory detail, it is worth mentioning that memory for color was low (near chance), and thus, this aspect of the data should be interpreted with some caution. Future investigations in this domain could work to bring performance for color context above chance. One way to do so is using an approach where there are multiple color contexts in which stimuli appear. Such an approach would reduce chance (e.g., having eight different colors would reduce chance to approximately 13%) making it more likely that participants would exhibit above chance memory for color context. Another possibility is to change the nature of the initial study phase task such that participants attended more to the color details as part of processing stimuli. For example, one such way is to ask participants to engage in visual imagery to create a mental image depicting the two words in the color in which the stimuli are presented. Such an approach is in line with past work demonstrating that visual imagery can be used as a strategy to facilitate encoding of information into memory (Hertzog & Dunlosky, 2006; Leshikar et al., 2012).

In this investigation, we found enhanced item and location context memory testing effects for materials encountered in lower compared to higher constraint test conditions, suggesting that testing conditions that provide fewer retrieval cues lead to enhanced memory for a variety of episodic details. We further found that both testing conditions (lower, higher constraint) improved memory on a final test compared to control conditions (restudy, study‐only), consistent with decades of past work on the testing effect (Adesope et al., 2017; Rowland, 2014). Overall, evidence in this report confirms that testing, or retrieval practice, is a powerful mnemonic tool that leads to improved memory for studied materials, and further that the nature of the test used in the re‐exposure phase has a large effect on the magnitude of the memory benefit arising from testing.

## AUTHOR CONTRIBUTIONS

**Konstadena L. Giannakopoulos**: Conceptualization; data curation; formal analysis; methodology; writing—original draft; writing—review and editing. **Matthew P. McCurdy**: Conceptualization; data curation; formal analysis; investigation; methodology; supervision; writing—original draft; writing—review and editing. **Allison M. Sklenar**: Methodology; writing—review and editing. **Andrea N. Frankenstein**: Methodology; writing—review and editing. **Pauline Urban Levy**: Methodology; writing—review and editing. **Eric D. Leshikar**: Conceptualization; data curation; formal analysis; investigation; methodology; project administration; resources; validation; writing—original draft; writing—review and editing.

## CONFLICT OF INTEREST STATEMENT

The authors declare no conflicts of interest.

## FUNDING INFORMATION

No funding was received for conducting this study.

### PEER REVIEW

The peer review history for this article is available at https://publons.com/publon/10.1002/brb3.3380.

## Supporting information



Supplementary Information

## Data Availability

Data will be made available upon reasonable request.

## NOTES

1 Twelve participants failed to show up for day 2 of the study, one participant received the wrong version of the experiment on day 2, and two participants did not follow instructions on day 2.

2 Participants were aware of the re‐exposure phase, but they were unaware of the final memory test given on day 2, as we have previously done (Giannakopoulos, et al., 2021). We chose to make this an incidental encoding task because past work has shown that testing effects tend to be reduced under incidental learning conditions (Pu & Tse, 2014). Because we wanted a “conservative” test of the effects of test constraint (lower, higher) on performance on a final test, we elected to use incidental study conditions in this experiment.

3 We performed two additional analyses on the item memory data. First, because we expected that the test conditions (lower, higher) would yield improved memory relative to the two control conditions (restudy, study‐only), we performed a planned comparisons directly comparing our test conditions against our control conditions. Results showed that item memory for the two test conditions was significantly higher than the two control conditions, *t*(46) = 10.95, *p* < .001, which is consistent with the typically found testing effect. Second, in our primary analysis of the item memory data, we used a measure of *conditionalized* item recall (e.g., proportion of correctly recalled items in the final test phase out of the total number of items correctly produced in the re‐exposure phase), but we also performed an analysis of item recognition using the uncorrected item recall proportions (e.g., unconditionalized data). We found significant differences among the conditions, *F*(3, 46) = 24.94, *MSE* = .195, *p* < .001, *η* ^2^ = .35. Follow‐up analyses revealed that the lower constraint test (*M* = .81) led to better memory compared to both the restudy condition (*M* = .76), *t*(46) = 2.45, *p* = .01, *d =* 0.36, and the study‐only condition (*M* = .69), *t*(46) = 5.27, *p* < .001, *d =* 0.77. Similarly, the higher constraint test (*M* = .83) led to better memory compared to both the restudy, *t*(46) = 4.42 *p* < .001, *d* = 0.65, and study‐only conditions, *t*(46) = 8.24, *p* < .001, *d =* 1.20. There was also a marginal effect between the two practice test conditions, *t*(46) = 1.81, *p* = .07, *d* = 0.26.

4 Similar to what we did for the item memory data, we performed two additional analyses on the location context memory data. First, because we expected that the test conditions (lower, higher) would yield improved location context memory relative to the two control conditions (restudy, study‐only), we performed a planned comparisons directly comparing our test conditions against our control conditions. Results showed that memory for the two test conditions was significantly higher than the two control conditions, *t*(46) = 6.04, *p* < .001, which is consistent with the standard testing effect. Second, in our primary analysis of the location context data, we used a *conditionalized* location context memory estimate (i.e., location hits/item hits), but we also performed an analysis of location context memory using the uncorrected location context hits (e.g., unconditionalized data). We found significant differences among the conditions, *F*(3, 46) = 14.09, *MSE* = .103, *p* < .001, *η* ^2^ = .23. Follow‐up analyses revealed that the lower constraint test (*M* = .25) led to better memory compared to both the restudy condition (*M* = .17), *t*(46) = 4.18, *p* < .001, *d* = 0.61, and the study‐only condition (*M* = .14), *t*(46) = 4.93, *p* < .001, *d* = 0.72. Similarly, the higher constraint test (*M* = .21) led to better memory compared to both the restudy, *t*(46) = 2.61 *p* = .01, *d* = 0.38, and study‐only conditions, *t*(46) = 4.31, *p* < .001, *d* = 0.63. There was also a marginal effect between the two practice test conditions, *t*(46) = 1.93, *p* = .06, *d* = 0.28, which is in line with our primary analyses reported in the main text.

5 Although our primary focus is on the memory component of these data, we performed analyses on the confidence ratings for both the location and color retrieval decisions. We include analyses of the confidence ratings in the Supporting Information.
