# Supplementary material for: Lower constraint testing enhances the testing effect for some contextual details but not others
Source: Brain Behav. 2024 Jan 9;14(1):e3380. doi: 10.1002/brb3.3380 (PMC10776958; doi:10.1002/brb3.3380)
Supplement: Supplementary file 1 — Supplementary Information [file BRB3-14-e3380-s001.docx]

Supplementary Materials

Confidence ratings analyses.

We ran a repeated-measures ANOVA on the confidence ratings collected during the final test phase for the location context measure. Across the four conditions we found a significant difference, *F*(3, 46) = 19.32, *p* < .001, $\eta^{2}$ = .30. Follow-up analyses revealed that the lower-constraint test (*M* = 2.95, *SD* = 1.24) was rated significantly higher than both the restudy condition, (*M* = 2.60, *SD* = 1.13), *t*(46) = 4.02, *p* < .001, *d* = .59 and the study-only condition, (*M* = 2.37, *SD* = 1.09), *t*(46) = 5.94, *p* < .001, *d* = .87. Similarly, the higher-constraint test (*M* = 2.76, *SD* = 1.18) was rated higher compared to the restudy control, *t*(46) = 2.96, *p* = .005, *d* = .43, and the study-only condition, *t*(46) = 5.23, *p* < .001, *d* = .76. Interestingly, there was no significant difference between the lower-constraint test and the higher-constraint test.

Additionally, we ran a repeated-measures ANOVA for the confidence ratings for the color context measure which resulted in a significant effect, *F*(3.46) = 12.01, *p* < .001, $\eta^{2}$ = .21. Planned follow-up analyses revealed that the lower-constraint test (*M* = 3.00, *SD* = 1.17) had higher ratings than both the restudy control, (*M* = 2.77, *SD* = 1,12), *t*(46) = 3.04, *p* = .004, *d* = .44 and the study-only condition (*M* = 2.61, *SD* = 1.12), *t*(46) = 5.28, *p* < .001, *d* = .77. Additionally, the higher-constraint test (*M* = 2.96, *SD* = 1.21) had higher confidence ratings than the restudy control, *t*(46) = 2.63, *p* = .012, *d* = .38 and the study-only condition, *t*(46) = 4.60, *p* < .001, *d* = .67. There was no significant difference between the lower-constraint test and higher-constraint test, *t*(46) = 0.46, *p* = .65, *d* = .07.
